# Vaccines of the Future: The Role of Inflammation and Adjuvanticity

**DOI:** 10.1155/2015/789595

**Published:** 2015-08-26

**Authors:** Cheol-Heui Yun, Luciana C. C. Leite, Aldo Tagliabue, Diana Boraschi

**Affiliations:** ^1^Department of Agricultural Biotechnology and Research Institute for Agriculture and Life Sciences, Seoul National University, Seoul 151-921, Republic of Korea; ^2^Institute of Green Bio Science and Technology, Seoul National University, Pyeongchang, Gangwon-do, Republic of Korea; ^3^Centro de Biotecnologia, Instituto Butantan, Avenida Vital Brasil 1500, São Paulo, SP, Brazil; ^4^Lombardy Region Foundation for Biomedical Research, Via Taramelli 12, 20124 Milan, Italy; ^5^Institute of Protein Biochemistry, National Research Council, Via Pietro Castellino 111, 80131 Napoli, Italy

With the increase of life expectancy worldwide, people expect a better quality of life, and vaccination is possibly one of the best tools for improving it. In addition to developing or improving vaccines for the elimination of infectious diseases such as malaria, tuberculosis and AIDS, the challenge of future vaccination strategies is both to improve efficacy of currently available vaccines for groups of people with frail immunity, such as the elderly or patients with chronic diseases, and to develop new vaccines for non-communicable diseases such as autoimmunity and cancer. More personalized or precise vaccination is one of the aims of future vaccines, taking into account age, gender, health and nutritional status, together with geographical/environmental conditions.

Vaccine efficacy is greatly enhanced by adjuvants. Adjuvants are agents or strategies that cause the initiation and generation of protective memory by inducing a mild innate/inflammatory reaction followed by the amplification of vaccine-specific adaptive immunity. Safety has therefore been a major issue with adjuvants, since adverse effects are hard to avoid and inevitable especially after a strong inflammatory reaction. For a long time, the only adjuvant approved for human use has been Alum (particulate aluminium salts), until the recent development and approval of new adjuvants such as monophosphoryl lipid A (MPL) and oil-in-water emulsions (such as MF59). It is clear that development of new safer adjuvants coincident with the improved design of their use will greatly influence the efficacy of modern vaccines. Novel technologies will allow us to achieve better and safer vaccination and protection through the induction of broadly neutralizing antibodies. Novel adjuvants/delivery systems for multiple antigens will be developed by structural vaccinology, reverse vaccinology, and nanotechnology. With biodegradable nanosized materials, we expect to maximize vaccine efficacy through better targeted delivery and concomitant adjuvanticity.

In this exciting period of reborn interest for vaccines, it is important that we investigate and revisit the mechanism of action of old and new adjuvants and provide insights for their practical use in vaccine formulations. Thanks to the impressive advancements in the knowledge of innate immune mechanisms, including activation of Pattern Recognition receptors, in particular Toll-like receptors (TLR), and the new concept of innate memory, novel adjuvants will be able to selectively activate one or more of these pathways in a controlled fashion, thereby achieving optimal efficacy and reducing adverse effects.

In this special issue, a number of papers will illustrate the most recent advances in the concept of adjuvanticity and controlled inflammation in achieving optimal protection and concomitant safety. E. Töpfer et al. will introduce the concept of innate memory and how this could be exploited for improving vaccine efficacy. A group of papers will then illustrate the most recent advances in exploiting TLR agonism for optimizing adjuvanticity and modulating the resulting immune responses (J. Bortolatto et al., M. Herbáth et al., T. Aoshi et al., and N. S. Daifalla et al.). Other contributions will describe new technologies for designing vaccine antigens with endowed adjuvanticity (L. D'Apice et al.), for exploiting extracellular vesicles to concomitantly obtain antigen delivery and adjuvanticity (J. H. Campos et al.), and for precise localization and delivery of plasmid DNA in veterinarian DNA vaccines (D. Dory et al.). A last group of papers deal with the key issue of efficacy and safety. S. Di Mario et al. discuss significant differences in the efficacy of two anti-HPV vaccines in naive versus infected women. A. T. Gunes et al. address the issue of possible effects of vaccination on triggering immune-related affections. Last but not least, D. Lewis and M. Lythgoe close the special issue by presenting a comprehensive platform of systems vaccinology for evaluating the inflammatory and reactogenic effects of adjuvanted vaccines, with the final goal of ensuring the optimal safety of future vaccination strategies.

It is our hope that the knowledge-based development of new adjuvanted vaccines, relying on the modulation of inflammatory responses suitable for the protection, will not only provide improved protection against new and re-emerging infectious diseases, but also enhance the quality of life of the human population.

*Cheol-Heui Yun* *Cheol-Heui Yun* *Luciana C. C. Leite* *Luciana C. C. Leite* *Aldo Tagliabue* *Aldo Tagliabue* *Diana Boraschi* *Diana Boraschi*

